# Integrating Quantitative Knowledge into a Qualitative Gene Regulatory Network

**DOI:** 10.1371/journal.pcbi.1002157

**Published:** 2011-09-15

**Authors:** Jérémie Bourdon, Damien Eveillard, Anne Siegel

**Affiliations:** 1 Computational Biology (ComBi) Group, LINA UMR 6241, Université de Nantes, Ecole des Mines de Nantes & CNRS, Nantes, France; 2 IRISA, Symbiose, INRIA Rennes-Bretagne-Atlantique, Rennes, France; New York University, United States of America

## Abstract

Despite recent improvements in molecular techniques, biological knowledge remains incomplete. Any theorizing about living systems is therefore necessarily based on the use of heterogeneous and partial information. Much current research has focused successfully on the qualitative behaviors of macromolecular networks. Nonetheless, it is not capable of taking into account available quantitative information such as time-series protein concentration variations. The present work proposes a probabilistic modeling framework that integrates both kinds of information. Average case analysis methods are used in combination with Markov chains to link qualitative information about transcriptional regulations to quantitative information about protein concentrations. The approach is illustrated by modeling the carbon starvation response in *Escherichia coli*. It accurately predicts the quantitative time-series evolution of several protein concentrations using only knowledge of discrete gene interactions and a small number of quantitative observations on a single protein concentration. From this, the modeling technique also derives a ranking of interactions with respect to their importance during the experiment considered. Such a classification is confirmed by the literature. Therefore, our method is principally novel in that it allows (i) a hybrid model that integrates both qualitative discrete model and quantities to be built, even using a small amount of quantitative information, (ii) new quantitative predictions to be derived, (iii) the robustness and relevance of interactions with respect to phenotypic criteria to be precisely quantified, and (iv) the key features of the model to be extracted that can be used as a guidance to design future experiments.

## Author Summary

Understanding the response of a biological system to a stress is of great interest in biology. This issue is usually tackled by integrating information arising from different experiments into mathematical models. In particular, continuous models take quantitative information into account after a parameter estimation step whereas much recent research has focused on the qualitative behaviors of macromolecular networks. However, both modeling approaches fail to handle the true nature of biological information, including heterogeneity, incompleteness and multi-scale features, as emphasized by recent advances in molecular techniques. The principle novelty of our method lies in the use of probabilities and average-case analysis to overcome this weakness and to fill the gap between qualitative and quantitative models. Our framework is applied to study the response of *Escherichia coli* to a carbon starvation stress. We combine a small amount of quantitative information on protein concentrations with a qualitative model of transcriptional regulations. We derive quantitative predictions about proteins, quantify the robustness and relevance of transcriptional interactions, and automatically extract the key features of the model. The main biological novelty is therefore the presentation of new knowledge derived from the combination of quantitative and qualitative multi-scale information in a single approach.

## Introduction

There have been a number of success stories in macromolecular network modeling during the last decade. Special attention has been paid to dynamical modeling approaches. Among a broad spectrum of strategies, qualitative models and their associated methods have played a central role, allowing modelers to investigate the full space of possible discrete behaviors of several regulatory networks. To that end, a variety of methods for qualitative modeling, analysis and simulation of genetic regulatory networks (GRN) have been proposed since the seminal works of Kauffman [1] and Thomas [2], [3] (see [4] for a review). As they rely on discrete representations of both time and variables, these methods share two main advantages: first, the space of possible states is finite (although possibly large), making it possible to hypothesize about the dynamics of biological regulatory systems despite the lack of kinetic information at transcriptional level. Second, regulatory networks can be built from local experimental observations or knowledge-based information (gene-gene or gene-protein interactions).

Although these approaches provide high-level insights into the functioning of gene networks, they often do not accurately reflect the real dynamics of GRN. Indeed, transitions between states in a GRN may exhibit a stochastic component as observed in [5]. This stochastic signal is closely related to population average behaviors [6]. Consequently, the dynamics of GRNs have a stochastic component which is difficult to observe in real time and to capture in discrete models. This has emphasized the need for probabilistic models and methods for analyzing and simulating GRN. Such probabilistic representations of gene networks are now widespread to complement discrete approaches. The Probabilistic Boolean Network (PBN) approach [7], [8] is one of these. Due to its flexibility and the fact that it can be inferred directly from data, it has been extensively studied over the last decade. In [9], finite state Markov chains are also proven to be useful in dealing with microarray data. It was established that the automatically reconstructed Markov chain gave rise to steady state distributions in accordance with some phenotypic biological observations. This suggests that Markov chain models are capable of mimicking biological behavior. More generally, Markov chain models are usually applied in the following way. First, a model that fits a given set of data is inferred [10], [11]. Then, steady state distributions are computed, giving access to biological information, as they reflect some expected phenotypes [8], [12]. In a final step, important product nodes are exhibited, as they control the steady-state distribution and the phenotype [5], [13], [14]. This latter task gives insights useful in designing new biological experiments, allowing both a better validation of the model and suggesting some therapeutic targets. Although those approaches are very efficient, they mainly rely on the quality of the network reconstruction process, that yields a two sides issue: inferring the “structure” of the gene regulatory network and computing transition probabilities that are consistent with the available data. In concrete terms, the lack of accurate observation datasets on the result of transition in a GRN usually makes the inference of the structure more accurate than the computation of the probabilities [5].

In a quite complementary way, [15], [16] have proven that adding a probabilistic aspect to already qualitatively validated discrete models may help in determining parameters of the qualitative model. To do so, the authors add a probabilistic dimension to a discrete piecewise affine model. They introduce unknown transition probabilities between two states as the ratio of volumes defined by the qualitative parameters of the system. The main novelty of their approach is that they compute the whole set of transition probability matrices leading to given qualitative attractors of the system, instead of selecting a precise matrix as the above-mentioned approach does. This approach allows them to exhibit relations between transition probabilities and important coefficients of the system such as synthesis rates. However, as they use an analytic description of the set of accurate probability matrices, their method is limited to small networks composed of two or three genes.

In the present work, we advance the idea of studying discrete knowledge-based transcriptional “intracellular” regulatory information given by qualitative models within a global probabilistic approach. The main novelty of our approach is that we compute the full set of probability transition matrices that correspond to quantitative “population scale” observations provided by protein time-series measurements. We rely on methods inspired by average-case analysis of algorithms theory [17], [18], making use of Markov chains coupled with transition costs to study statistical properties of pattern matching issues. We design a probabilistic framework allowing population scale observations to be integrated into a qualitative gene expression network assumed to be shared by several individual cells. Our approach should therefore be considered as a bridge between purely discrete modeling approaches and probabilistic simulations. We introduce three main novel features: first, we rely on a strong asymptotic property of Markov chains to fully describe the set of all possible weighted probabilistic networks matching with protein time-series observations. Second, we overcome computational problems as we drastically reduce the size of the model by focusing on slope changes (switch from a variable increase to a variable decrease, for instance) instead of changes in product levels. Third, we develop numerical methods to incorporate a set of suitable Markov chains – all those matching the numerical observations – rather than a single Markov chain that cannot be uniquely determined from the few quantitative observations we have at hand. These three novelties allow us to increase the robustness of our approach while reducing the set of data required to perform the analysis. Concretely, our approach involves first computing a discrete (non-deterministic) description of possible succession of slope variations. This can be deduced from knowledge-based transcriptional information, *i.e.*, either a logical graph or a qualitative event succession like those observed in novel generations of microarrays [19]. This provides us with a graph of transcriptional event transitions. The transcriptional events, arising on the scale of an individual cell, affect the protein concentrations, observed on a population scale. These two scales are related by adding an *impact cost* for each transition over a given protein concentration. This cost is easily deduced by fixing an arbitrary “natural” degradation rate and by applying an equilibrium principle as follows. Intuitively, in the absence of any information – when all the transition probabilities are chosen to be uniform – the expected protein concentrations will be constant. The next step consists of numerically determining the set of transition probability matrices that fit a global quantitative observed outcome. As an example, we expect the model to fit the time-series quantitative observations of the mean concentration of a single protein over a cell population - in this paper we focused on carbon starvation response in *Escherichia coli*. We have combined theoretical properties of Markov chains - inspired by symbolic dynamics - with reverse-engineering methods (local inference methods) to describe the full space of weighted Markov chains having the appropriate topological structure and whose global mean outcome fits the time-series curve. Then we investigate the geometric structure of the space of Markov chains to derive biological properties of the system: we derive a ranking of gene interactions with respect to their importance in achieving the considered protein variations. Such a classification is confirmed by the literature. We also accurately predict the quantitative time-series evolution of several non-observed population-cell protein concentrations using only knowledge of discrete gene interactions and very few quantitative observations on a single protein concentration. According to our modeling framework, variations in protein quantities appear to be driven by the dynamical behaviors, qualitatively described, that occur underneath at the gene regulatory scale.

## Method

### Main features

**Figure 1:**
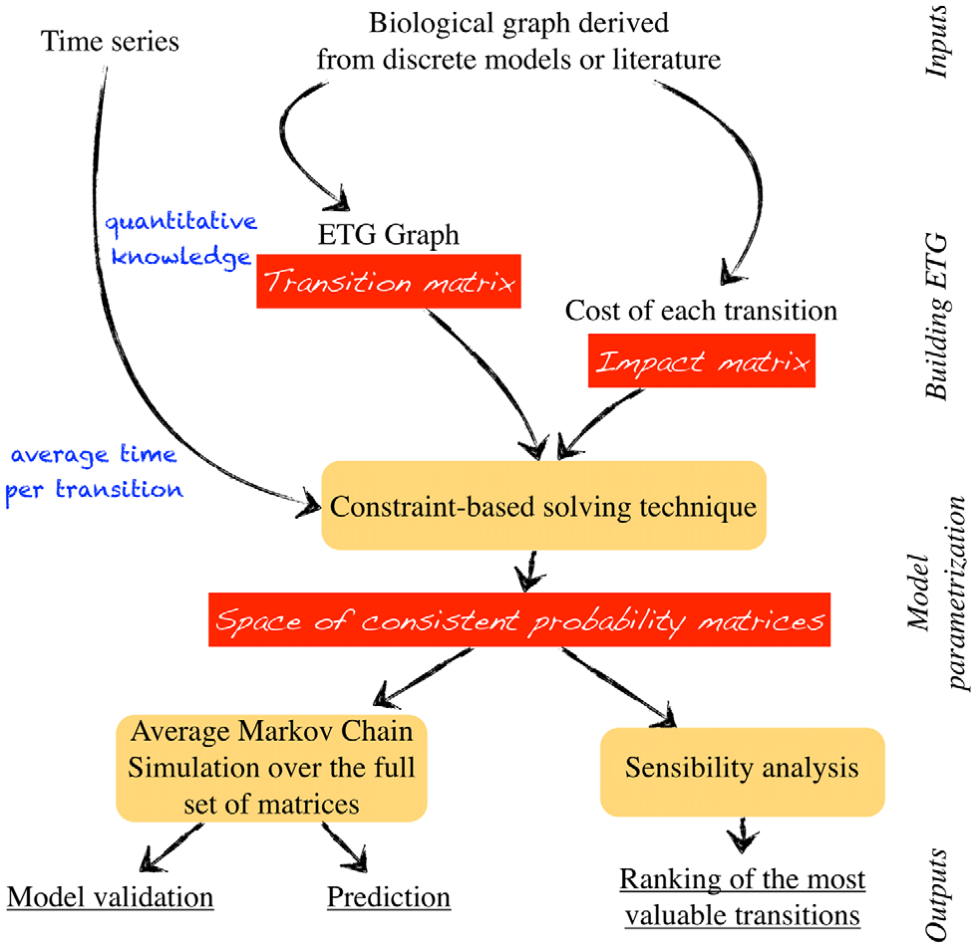
Flowchart of the Event Transition Markov chain modeling protocol.

As a major modeling contribution, and in the light of the above assumptions, this paper establishes a relationship between the concentration time series ( *i.e.*, quantitative knowledge) and the qualitative behaviors of the biological system, as modeled by genetic regulatory networks. To that end, two matrices are considered (see Figure 1). Note herein that an exhaustive illustration of following features is proposed in the end of the Method section. The first matrix describes an *event transition Markov chain* which constitutes the core of the model. It depicts the probabilities (latent variables of the model) that the system will switch from one qualitative “basic behavior” to another, where a qualitative basic behavior means a constant slope for the variation of a product. The structure of the matrix is determined by the current extent of our knowledge of what regulates the system. Its numerical coefficients stand for the mean ratio of trajectories of the system that may cross a given transition. Our reverse engineering method aims at computing these numerical non-zero coefficients. As a companion matrix to this event description, a family of *impact matrices* is built for each protein involved in the system. Given a protein 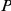, the corresponding impact matrix will describe the global outcome of each transition between two events – corresponding to an arrow of the Markov chain – over the concentration of the protein 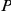. By way of example, if we assume that the system goes through a transition that activates the mRNA production of a gene 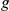, the effect (or “impact”) of this event may be modeled by an increase in the production rate of the protein 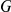 encoded by 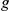, say 20%. Additionally, the effect of this event on all other proteins in the system may be modeled by a decrease in the production rate, a free parameter that we fix to 5%, since they undergo a natural degradation process and are not affected by the event transition. As detailed hereafter, the exact rates that are used are computed so that active and passive degradation have the same average impact during a random process. With these two matrices at hand, average-case analysis properties of Markov chains reveal a relationship between the event transition matrix, the impact matrices and the quantitative evolution of a protein concentration under given stimuli, allowing to establish some relations between observable variables of the model (the observed growth ratio of given proteins) and the latent variables of the model. Roughly, the time-series concentrations of a given protein make it possible to recover the main eigenvalue of the event transition matrix, which can be reformulated to infer times-series concentrations of other proteins, as well as global properties of the system.

### Average impact of a Markov chain over an accumulation rule

A *Markov chain* is a random process for which the next state depends on the current state only. It is described by a graph over the set of nodes 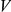, and edges labeled with probabilities in 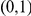. Likewise, the random process can be described by a *transition matrix* 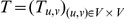. The Markov chain is described as *minimal* when this matrix is aperiodic and irreducible meaning that for sufficiently large 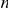 and all vertices 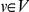, there exists an 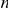-length cycle including 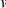. A *stationary state* of the Markov chain represents a numerical distribution of the nodes that does not evolve anymore, which corresponds to the eigenvector of the matrix 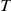.

The main goal is to estimate the quantitative asymptotic impact 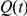 of the Markov chain on a biological product quantity or a generic yield. Biologically, such a quantity is any of the phenotypic measurements that is impacted by the gene regulatory network, *i.e.*, any experimental bio-product concentration that might be inferred from either a cell growth rate or a protein concentration encoded by a gene that belongs to the system. To this end, an *impact matrix* 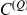 is linked to the transition matrix 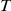 of the Markov chain. The impact matrix is the same size as 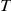. Zero-coefficients in 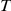 yield zero-coefficients in the impact matrix. Coefficients of the impact matrix are positive real values that describe the estimated cost of a transition on the change in the phenotypic quantity.

Impact matrices simulate the effect of a Markov process over the global quantity 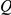 as follows. Let 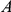, 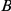 be two nodes of the Markov chain connected by an edge 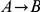. Let 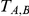 denotes the probability of this transition and 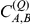 its impact. The *elementary cost* of the transition 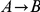 over the quantity 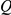 is defined as 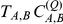. The induced *elementary cost matrix* is denoted by 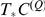. The quantity 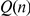 is then said to evolve following a *multiplicative accumulation rule from an initial distribution* 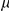. Its mean value at time 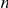 – *that is, after* 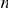 *iterations of the Markov process* – ( *i.e.*, the average of the costs of all trajectories of length 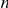) is strongly related to powers of elementary cost matrix, that is 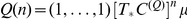. In other words, to compute the mean value of the quantity at step 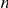, the elementary cost is multiplied along all paths of length 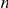 – therefore introducing 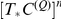. Each path is weighted with the probability of starting from its initial node – information given by 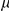. The final impact is given by the sum of all these quantities – therefore multiplying by 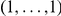. In particular, as detailed below, such a multiplicative accumulation rule is useful to model the burst effect of a gene regulatory network on a metabolic scale, in which a single mRNA stochastically transcribed produces a burst of protein copy numbers [20]–[23].

When a Markov chain is fully determined and when an impact matrix is given, simple linear algebraic computations allow to calculate the growth rate of the corresponding quantity. The added value of a multiplicative law over a Markov chain relies on its asymptotic behavior, that is proved to be exponential, as stated in Theorem 1. More precisely, a multiplicative accumulation rule follows an explicit log-normal law with explicit mean, variances and estimation of error terms. All these characteristics, such as the growth rate 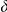 of the exponential, are related to dominant eigenvalues of the elementary cost impact matrix 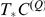. It should be noted that when the Markov chain reaches a stationary state, the accumulation law itself enters a *permanent regime*, where its exponential rate is fixed. The error term is also exponential, but with a much smaller growth rate, ensuring that the stationary state of the Markov chain is quickly reached.

### Theorem 1

*(Average case analysis theory for accumulation rules) Let* 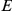 *be a minimal Markov chain with transition matrix* 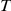 *. A multiplicative accumulation rule* 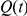 *with impact matrix* 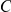 *asymptotically satisfies a* 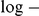 *normal law with mean and variance*

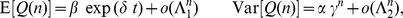
where 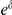 is the dominant eigenvalue of the elementary cost matrix 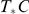. The other quantities express by means of a generation of the elementary cost matrix, 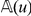 defined by 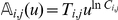. More precisely, 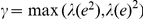 express by means of the dominant eigenvalue 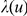 of 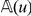, 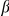 and 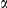 are constants corresponding to the dominant eigenvectors of 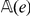 and 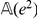. There exists 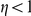 such that the error terms 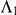 and 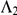 verify 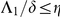 and 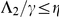.

Here, the minimality assumption restricts applications to a biological process such that 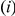 its underlying Markov chain is aperiodic and irreducible; and (ii) for every considered cost matrix, there exists at most one aperiodic trajectory (meaning that the cost evolution is aperiodic through times for this trajectory). Note that in the present work, these assumptions are those that will most restrict the biological referential. For instance, biological systems that display oscillatory behavior are outside the natural range of the approach. Nonetheless, one may overcome this weakness by modeling an input with oscillatory behavior and modeling the steps of the dynamics with independent Markov chains. This modeling device is particularly useful when one aims at modeling the circadian system. For a better illustration, please see below how to build such a Markov chain that describes the behaviors of a gene regulatory network.

### Reverse engineering of a transition matrix from impact accumulation rules and growth rates

Given a set of impact rules and assuming that they all follow accumulation rules, optimization techniques were used to infer a Markov chain fitting all available experimental results – the growth rate of several biological quantities. The identification process was divided into two optimization problems. First, in the exact case, a Markov chain is computed which minimizes the euclidean distance between the growth rates 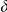 and 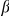 – see Theorem 1 above – of every impact rule associated with the Markov chain and the objective numerical values provided by the experimental results at hand. Local search algorithms are well suited to such an inference task (see [24] for a review). Here, it is necessary to develop an ad-hoc local search algorithm capable of handling eigenvalues that have only an implicit definition.

In order to take experimental errors into account, we considered a second optimization problem, in which the objective values were defined by an interval of validity. Our goal was to infer a Markov chain such that the growth rate of every impact rule belongs to its objective numerical interval, allowing some sets of valid Markov chains to be defined. These sets were approximated by using a polyhedra, defined as follows. First the local search algorithm was used to find a Markov chain whose growth rates were close to the middle of every objective intervals. This Markov chain defines a point, hereafter called the source point in the sequel, inside the solution set. Some points on the boundary of the solution set were then identified by setting a random direction and using a dichotomy method to find the intersection between the boundary and the line, starting from the source point with the expected random direction. As shown in the results section, the volume provides particularly meaningful information. In both cases, sensitivity analysis was performed by considering the following definition. The function 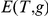 was introduced, standing for the Euclidean distance between the growth rate of all impact rules and their objective numerical values. The *sensitivity of a transition* 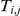 is then defined by the 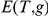 modification, in percent, when 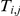 is modified by 1%. Note that it is closely related to the partial derivative according to variable 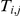 of the function 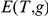. The higher is the sensitivity of a transition, the more sensitive is the overall score to small variations of this variable.

### Event transition Markov chain associated with a gene regulatory network

The previous theoretical framework can easily be adapted to the biological regulatory networks that display discrete dynamics [25]. Products of the system are gathered in a set 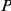 and a relevant Markov chain summarizes the dynamics of the system. In order to handle computational issues of reverse engineering, the focus is on shapes of trajectories instead of graph states, formalized as follows.

The main component of the modeling operation are transcriptomic events, *i.e.*, elements of 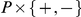. They describe the possible slopes in the variation of a bioproduct during a time unit (i.e. increasing or decreasing). For instance, 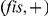, also denoted by 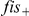, stands for the increase in the transcriptional activity, or mRNA production, of the gene 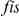. The two events occurring over a product 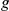 are denoted by 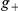 and 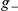. It is sometimes useful to add some supplementary biological events such as a complex formation, when the information is available. This increases the accuracy of the model. The *Event Transition Graph* (ETG) encodes the possible successions of events. Its nodes are given by the set of events. An event 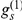 targets 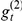 if, in at least one trajectory of the system, 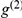 varies with the slope 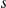 and then 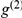
's slope changes to the sign 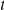. This graph may be derived easily from a state transition graph such as those produced by logical asynchronous multivalued Thomas mode piecewise linear models [26].

An *Event transition Markov chain* is an event transition graph endowed with a matrix probability 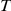. Biologically, considering a Markov chain means considering an average behavior of the system over a set of different cells. Since the focus is on events only (i.e. successions of changes in the slope variations of products) instead of states, the stationary states of the Markov chain correspond to cell populations where the proportion of cells with increasing/decreasing transcripts is fixed. Therefore, the stationary states of Markov chains do not correspond to stationary states of the biological system (where all transcripts have a stable concentration). In order to avoid misunderstandings, a stationary state of an event transition Markov chain is called a *permanent regime*.

The *Initial state* of the Event transition Markov chain depends on the biological process that is studied. Assuming that the cells within a population are not synchronized suggests that the initial distribution of events in the system is uniform. If the cells are forced to be synchronized at an early stage of the experiments, a dedicated initial state describing the forced condition must be taken into account.

### Multiplicative impact matrix of the Markov chain over the production of each protein

It was pointed out that the evolution of one – or several – protein concentrations resumes a multiplicative phenotypic impact of the gene regulatory network [21], [23]. The multiplicative assumption was considered as relevant since the protein concentrations in a single cell follow standard evolution laws which are of exponential nature, similarly to the behaviors of systems governed by multiplicative laws [27]. Let 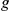 be a gene in the system at hand and 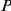 its encoded protein. The impact matrix 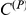 describes the impact of the event transition Markov chain on the protein production. To define this matrix, an *active impact scale* 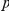 and a *passive impact scale* 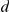 must be introduced. If a given transition impacts a given gene via its mRNA production, we assume that its encoded protein production increases or decreases by the scale 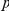. Otherwise the protein rate is assumed to decrease via its natural degradation by the scale 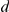. Formally, let 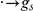 be an edge in the Markov chain (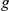 can be any product and 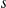 is either 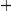 or 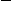). Reaching state 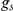 means that the activity of gene 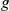 changes leading to an *active production or degradation* of its associated protein 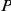. During all other transitions 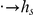, where 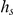 does not encode the protein 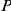, the system undergoes a natural degradation of protein 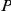. The production and degradation rate values are chosen as follows. The *passive effect* 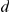 is set as equal to 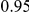 ( *i.e.*, a natural degradation of 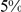). The *active degradation coefficient* is defined according to the following equilibrium rule. Let 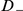 (resp. 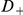) be the set of all events associated to an active degradation (resp. production) of the given protein. We first fix all the transitions to be uniform ( *i.e.*, all the probabilities of leaving a given state are equal), and denotes by 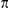 the steady-state distribution of the associated Markov chain. Protein 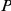 concentration is stable if
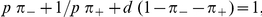
where 
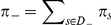
 and 
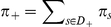
. This defines a degree two equation. Simple arguments prove that this equation has only one solution smaller than 1 that is assigned to 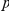. The *active production coefficient* is then defined as 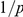, the inverse of the active degradation coefficient. Eventually, the impact matrix associated to the protein 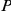 is fulfilled thanks to the passive effect rate and the passive and active degradation rates.

### Inferring growth rates from protein observations

As the approach is dedicated to prokaryotic systems, a linear relationship between gene activities and their protein concentrations is assumed. This induced a standard evolution law to describe the quantitative evolution of the protein concentrations in the system in accordance with the qualitative events as described by the event transition Markov. More precisely, it was assumed that, as with other modeling studies [23], [27], a protein concentration evolves according to a succession of exponential laws 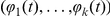, with 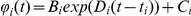. The cutting points 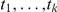 are obtained using the available experimental data. The meaning of this succession is that the protein concentration at time 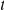 is 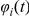 if 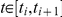. Then, for each 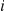, 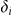, 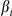 and 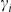 expresses by
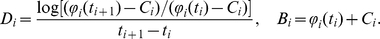
It can be noted here that the concentration of a protein that is only degraded tends to 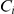, which is its basal concentration. Assuming it to be null leads to simpler formulas for 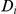 and 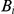.

According to the hypotheses discussed below, we assume that the protein concentration 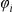 follows a multiplicative accumulation rule 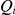 in each time interval 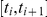. Let 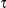 be the mean duration of a transition. In the permanent regime of 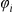, which is reached very quickly, the relation 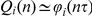 holds. According to Theorem 1, this equation implies that the product 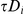 is nothing but the dominant eigenvalue 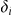 of the elementary cost matrix of 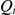. Additionally, 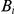 introduced below equals the constant 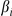 introduced in Theorem 1.

Taking all into account, the growth rates 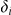 and 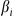 required to apply our reverse-engineering methods described below, can be calculated from the protein concentration shape as soon as the mean duration time 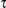 of a translation has been estimated. To that end, it is assumed that the duration is independent from the studied dynamics, allowing it to be computed from experimental knowledge on passive degradation. We introduce 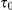 the shortest half-life of amino-acids of the protein of interest – usually available in the literature. According to the N-end rule, as depicted in [28], fixing a passive degradation rate of 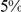 entails that 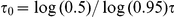, which allows an explicit computation of 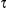 and completes the inference of growth rates.

### Illustration of the method on a two gene network

**Figure 2:**
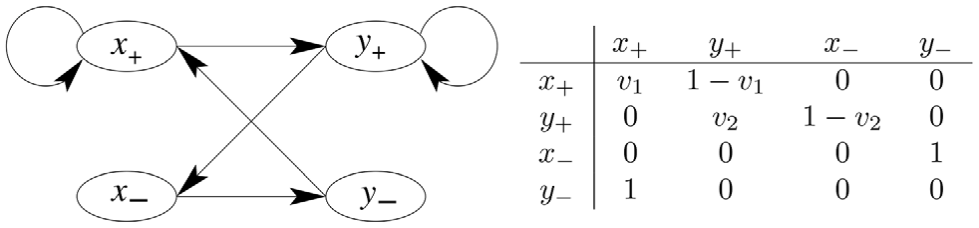
Event Transition Graph composed of 2 genes (left) and its corresponding probability transition matrix (right), that includes two unknowns 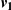 and 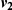.

For the sake of clarity, we propose to illustrate now the modeling method when applied on a simplistic Event Transition Graph (core model). It is composed of two genes that monitor four events as depicted in Figure 2. The graph is also depicted using a transition matrix in which one adds two unknowns (latent variables) for describing a Markov chain: 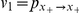 and 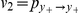. To solve the problem in a biological context, one then considers the two following complementary informations:

- *Costs per transition (free parameters)*: Assuming a passive degradation rate (free parameter) of 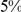 and applying the above equilibrium rule, the active degradation rate for both protein X and protein Y equals 0.882 (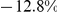) while the active production rate equals 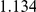 (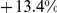). See Supplementary Text S1 for the matricial description. Here we assume that the time unit is one iteration of the Markov chain. In some more general cases, the definition of time units is tricker as mentioned above.
- *Fictive experimental knowledge (observable variables)*: For illustration and as tutorial, one considers that the protein X relative quantity or concentration, is multiplied by 100 in 100 iterations or time units ( *i.e.*, two measures points are thus 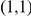 and 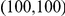, which defines an asymptotic growth rate equals to 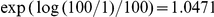).

**Figure 3:**
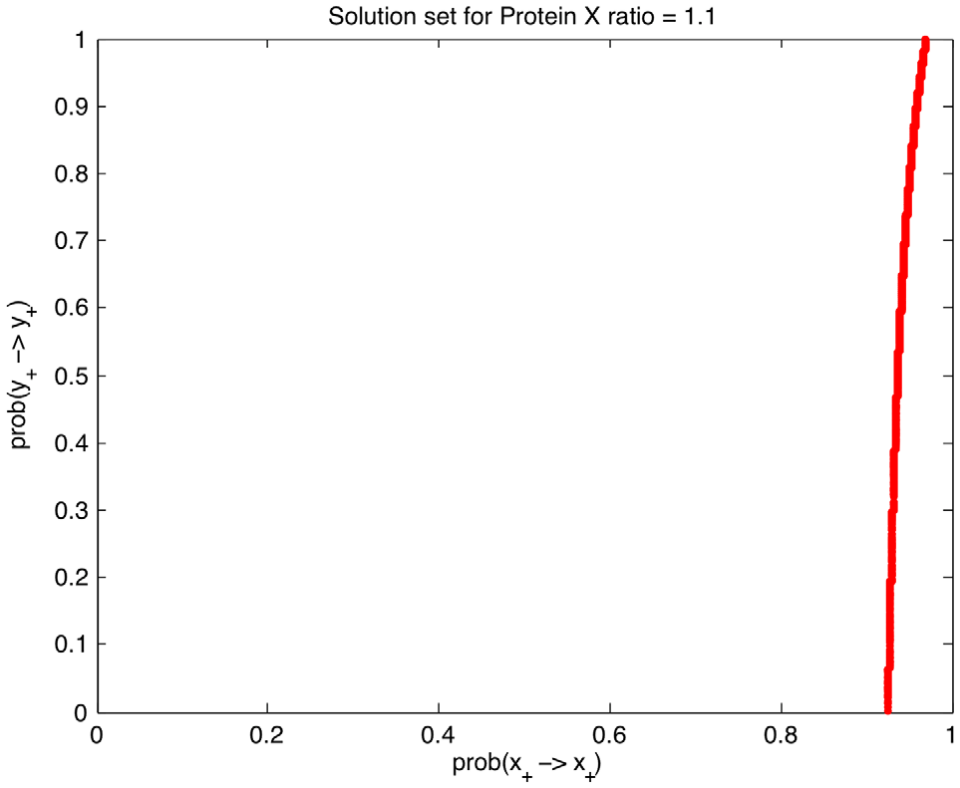
Set of probabilities that satisfy the constraints for the Event Transition Graph depicted in **Figure 2**.

**Figure 4:**
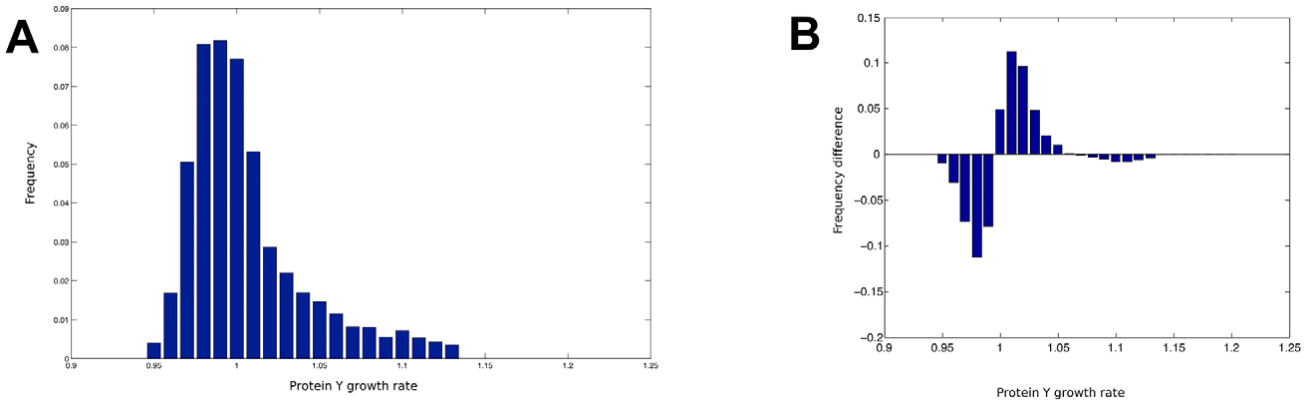
Comparison of the protein Y growth ratio in two different situations. (A) Distribution of the Y protein growth rate estimated from probabilities randomly picked; (B) Difference of the distribution described in (A), and the distribution of protein Y growth rate estimated from 10 000 combinations of probabilities that satisfies the constraints of the ETG model that depict the interactions of two genes.

These informations are then used to infer 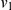 and 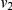 and relative probabilities. The inference task is performed by an adhoc matlab script (The complete package and its corresponding tutorial are available in http://pogg.genouest.org). As a general result, several combinations of probabilities satisfy the given constraints. They are depicted in Figure 3. Emphasizing a unique set of probabilities is therefore not possible. Unlike other Markov-like techniques, the Event Transition Markov chain models the impact of the Markov chain behaviors over the production of each protein of the system. We are thus able, for each combination of probabilities that satisfies the constraints, to estimate the protein growth rates in the permanent regime. Indeed, one can describe the distribution of Y protein growth rates for 10,000 probability combinations that satisfy the constraints (Figure 4(A)). This distribution is obviously sensitive to the probabilities. For illustration, the distribution of the protein Y growth rate for 10 000 probability combinations picked randomly is different, as attested when one depicts the difference of random and constrained distributions of Y protein growth rates in Figure 4(B), illustrating the close relations between protein X and Y concentration evolutions. Computing the distribution is not an easy task when one considers more than 3 genes or 6 events. In practice, we then overcome this problem by estimating the mean of each growth rate ( *i.e.*, 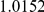 (prediction) in the case of the Y protein growth rate as presented above), instead of each growth rate distribution. This provides some accurate predictions of protein concentration evolutions.

## Results

**Figure 5:**
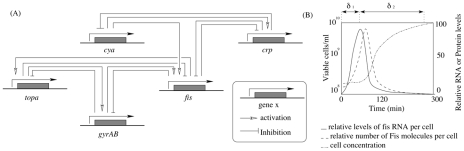
Biological information concerning *Escherichia coli* carbon starvation system. (A) represents interactions between genes involved in the regulatory network (adapted from [29]). (B) shows quantitative variations of macromolecules of interest (based on [30]). Note the linear relationship between *fis* mRNA and Fis protein productions that allows to infer protein product behaviors based on the gene regulatory network.

To illustrate the accuracy of the use of Event Transition Markov chains in a biological context, we propose now to focus the Event Transition Markov chain approach on predicting the behavior of protein concentrations during a period of bacterial stress. D. Ropers and collaborators model the growth phase transition of *Escherichia coli* after a period of nutritional stress [29]. In particular, their model shows the move from an exponential growth state to stationary growth during a carbon starvation stage. This elegant *“switch”* is evidenced at gene regulatory level with implications at phenotypic level. This model is based on the qualitative results available in both the literature and gene regulatory experiments as performed by the authors (see Figure 5). Furthermore, the proteins encoded by the genes that interact within the model have been well researched by independent studies [30], [31]. This provides partial quantitative information that may be introduced into the qualitative model.

### Event transition graph

**Figure 6:**
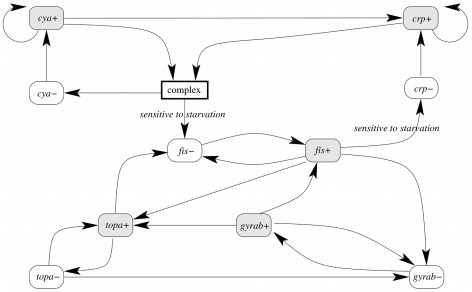
Even transition graph of the genes regulatory network of carbon starvation response in E. coli. Each component represents an active event that concerns a gene product (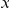), either its increase (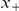) or its decrease (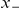). Arrows between events depict the active effect of one event on another. Two transitions are absent when the system is subject to carbon starvation.

The original model [29] is given as a system of piecewise affine differential equations. It contains 6 genes and 37 constraints over inequalities and thresholds. This yields a state transition graph of 912 qualitative domains. The corresponding Event Transition Graph was automatically computed by applying the definition introduced in the method section and detailed in Supplementary Text S2. The resulting graph, composed of 22 edges and 11 nodes, is depicted in Figure 6. Note that for the sake of clarity, we manually introduced a component named “complex” that summarizes the effect of cAMP metabolite as depicted in [32]. This node, in accordance to the original model [29], stands for a complexation of the Crp and Cya proteins and the carbon starvation signal. Following our formalization, this component is thus a natural product of 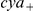, 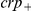 and the signal component. Although the event transition graph roughly summarizes the behaviors of the original qualitative model, it still highlights the major biological properties by its reading. For illustration, the repression of the *crp* gene by the Fis protein [33] is depicted by an active effect of 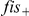 on 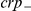. However, the information about *crp* controlled by two distinct promoters is lost.

### Event transition Markov chain: Impact and transition matrices

As detailed above in the method section, we computed the impact matrices based on bacterial protein production growth rates. This setting appears to be suitable since *E. coli* can be viewed as a multi-scale system. Indeed, the change in protein concentration shall be considered as a protein scale amplification of events that occurs at the transcriptomic scale that are depicted as protein burst by experiments [20]–[22]. By way of illustration and following the equilibrium rule defined above, in the impact matrix over the Fis protein, the concentration of Fis, denoted by 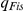, undergoes a 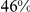 increase for each transition targeting 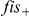. It suffers from a 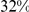 decrease for all transitions targeting 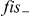. Finally, it goes through a 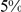 decrease for all other transitions, reflecting a natural degradation for Fis (see Supplementary Text S2 for a complete description of the impact matrix). This depicts the Event Transition Markov chain.

**Figure 7:**
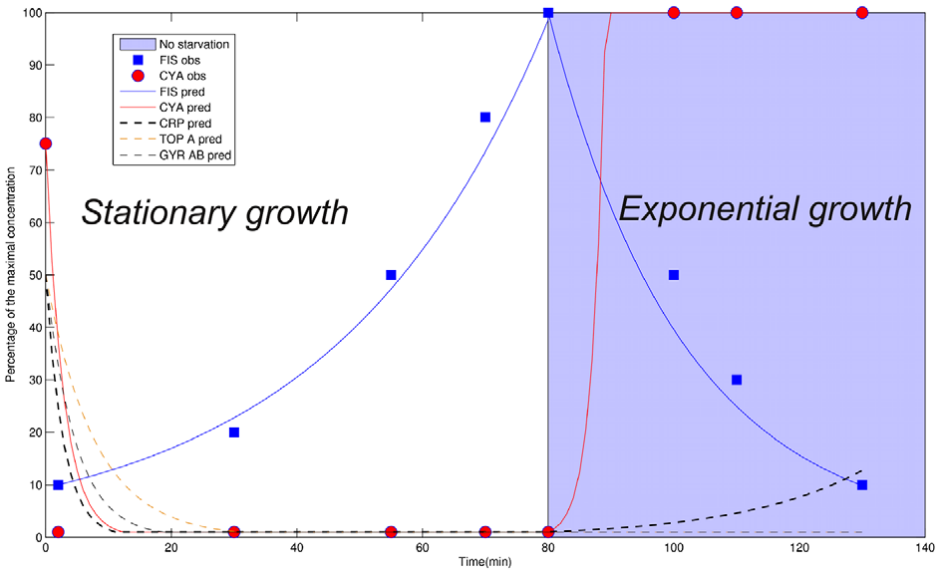
Simulations of changes in bacterial protein concentration during both stationary and exponential growth phases. The corresponding probability matrix is estimated in the stationary growth condition based on three experimental data for the protein Fis. After 80 minutes, the signal of carbon starvation manually switches from 1 to 0, emphasizing a switch from starvation to non-starvation conditions, which leads respectively to a stationary and an exponential growth phase of the bacterial population. Experimental data are marked with dashed lines, whereas computation results are depicted using plain lines for the five proteins of interest (Fis, Cya, Topa, GyrAB and Crp).

We used quantitative information about changes in Fis protein concentration to reverse-engineer the transition matrix. Experimental evidence [30] shows that the Fis concentration multiplies by 10 in 80 minutes, during the stationary growth phase (i.e. carbon starvation conditions) and then decreases in the exponential phase (see Figure 7 and Supplementary Text S2 for details). Therefore, the protein concentration curve was approximated by two successive steps 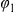 (stationary phase, from 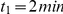 with 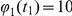 until 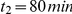 with 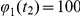) and 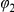 (exponential phase, from 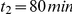 with 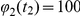 until 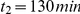 with 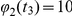). The shortest half-life of amino-acids of the protein of interest is estimated as 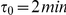 by the literature [28], leading to a mean transition duration of 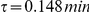. Applying our inference growth rate procedure – see method section – resulted in the computation of the growth rates for both the accumulation rules corresponding to the stationary phase (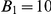, 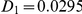, *i.e.*, 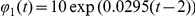) and the exponential phase (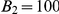, 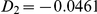, *i.e.*, 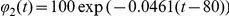). Then, the reverse-engineering approach using 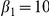, 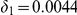, 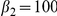, 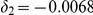 (see Method section) produced a probability transition matrix 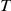 that fits the protein growth rates in both stationary and exponential growth phases. By repeating several times this procedure, one obtains a sampling of the set of all probability matrices that fits the given experimental protein growth rates.

### Asymptotic behavior of the system

Using the transition matrix of the Event Transition Markov chain, we perform several simulations on protein concentrations, as impacted by the gene regulation network. First, the transition matrix was coupled with impact matrices on proteins Fis and Cya to simulate their permanent regimes during the stationary phase. Then, after 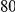 minutes, it is assumed that the exponential phase is initiated, inducing a change in the structure of the gene regulatory network. This change takes place by adding 2 transitions from the “signal” box on the Event transition Markov Chain which activates 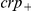 and the “complex” compound. Because of the given initial conditions during the exponential growth phase, these transitions were neglected, but not in stationary phase conditions. Then, based on the same matrices (impact and probability transition), new simulations are performed on the evolution of Fis and Cya concentrations. Figure 7 depicts the predicted variations of the Cya and Fis proteins during both phases.

Compared to the available independent experimental results [30], [31], the simulations and experiments are overall significantly similar according to a Pearson correlation test. The transition matrix allows us to compute the quantitative behavior of Cya in both stationary and exponential phases. Based on sparse information about Fis only, the predicted Cya behavior is consistent with the experimentally observed behavior (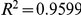, *p-value* 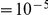) [31], which is a quantitative validation of our model. Notice herein that we also predict the complete time series of Fis (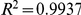, *p-value* = 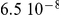), which confirms the exponential growth rate assumption. As a complementary result, the system remains for only a short time in the transient regime ( *i.e.*, the error made herein when one computes the mean is significantly lower than 1% after 7 minutes, or 20 iterations of the Markov Chain), which backs up our assumption of studying this microbial system in permanent regime in both growth conditions. This confirms the usefulness of our modeling approach for this specific biological system.

### Automatic classification of key gene interactions

In addition to the prediction feature, properties of the Markov chain provide insights into biological system behavior. According to the inference process, the proteins Cya and Crp have the same predicted behavior, as *a posteriori* confirmed by [34]. Furthermore, the sensitivities associated with the transitions of the Markov chain also represent an appreciation of the impact of a given biological compound. In particular, this demonstrates that, in stationary growth phase, 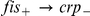 transition is highly constrained. Interestingly, this transition implicitly monitors the CAMP-CRP complex that controls the metabolism of alternative carbon sources [33]. It is closely related to ability to the bacterial system to switch between both growth phases in function of the carbon starvation. Furthermore, Schneider and co-workers [35] suggest that *fis* is involved in a fine tuning of the homeostatic control of DNA supercoiling. A small change in the supercoiling drastically affects the expression of the gene *fis*, which is in total agreement with the constraints extracted from the Event Transition Markov chain. We performed a similar analysis over the whole system ( *i.e.*, in both stationary and exponential growth conditions). The most sensitive transitions are reported in Table 1, in which we detail the biological meanings of such interactions. Not surprisingly, *fis* regulation is one of the corner stone genes of the system, but it might be a natural consequence of the inferring process in our modeling approach. However, with no specific transition matrix inference, *gyrAB* also emerges as one of the most, if not the most, important gene of the microbial system. Implicitly, this confirms the usefulness of the DNA topology for *E. coli* under carbon starvation conditions.

**Table 1:** Summary of the most important transitions of the system according to their sensibility measure.

Transition in ETG	Sensitivity	Biological significance	Ref.
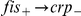	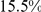	control of CAMP-CRP complex	[33]
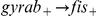	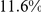	*fis* regulation controlled by the DNA supercoiling level	[37]
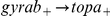	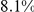	Topoisomerase I regulation by the DNA supercoiling	[38]
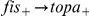	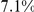	Homeostatic control of DNA topology	[35], [39]
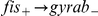	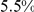	Homeostatic control of DNA topology	[35], [39]
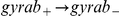	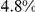	*gyrAB* expression regulation by the DNA supercoiling	[35]

## Discussion

Our purpose was to illustrate the strength of coupling Markov models together with accumulation rules to study the dynamics of a gene regulatory network, by focusing on its effects at a larger scale – the quantitative protein scale. We assumed that the production of a protein by a gene that belong to a regulatory network, follows a multiplicative accumulation rule. This implies that a permanent distribution of the protein system will be reached in a very short time. In such a regime, each protein concentration follows an exponential dynamic. The permanent regime may be modified by external events, inducing a short transition to another permanent regime. This paper details why observing such a permanent distribution – possibly several – at the protein level allows us to recover the main probabilistic law that governs the gene regulatory network. The law is thus described by a Markov chain over the succession of transitions at the transcriptomic scale. Very general properties of this Markov chain – average case analysis (see Theorem 1) – allow us to infer the Markov chain from a variety of heterogeneous information, such as qualitative behaviors based on existing models and partial quantitative data. We proposed an efficient algorithm based on this average case analysis to infer the Markov chain. In this method, it must be emphasized that the fundamental interest is to focus on transitions between biological events (slope variations of products during a time unit) instead of state variation as proposed by other state-of-the-art methods. Indeed, this abstraction of the system is required to reduce the size of the Markov chain in order to achieve the inference process.

Having determined this Markov chain allows us to study the main asymptotic properties of the dynamic system: identifying the main transitions implied in the permanent regime and sorting the relevance of transition patterns. All these predictions may be quite easily checked with additional experimentation. Conversely, experimentation allows refinement of the Markov chain inference process. Taken together, mixing the properties of a Markov chain with accumulation rules, provides a tool to determine the quantitative and asymptotic properties of a dynamic system.

For illustration and validation purposes, we computed a Markov chain for the event transitions of the *Escherichia coli* system in the carbon starvation. The computations were performed by using a gene regulatory network of this process and quantitative data about protein Fis production during the stationary phase. Our predictions of the behavior of Fis during the exponential phase and of Cya protein changes were confirmed by independent experimental observations, which emphasizes the ability of our approach to spread partial quantitative information through an Event Markov chain built from qualitative models. Moreover, our results produce various emerging properties such as (i) the sensitivity of a specific transition within the Markov chain or (ii) the quantitative prediction of gene products that are not directly optimized during the simulation. All these features reinforce our interpretation of the global quantitative behaviors of the natural system as modeled.

From a technical viewpoint, the main interest of this approach is as follows: it is not necessary to build quantitative differential dynamic systems that need accurate and complex parameter estimations. Our method uses the results of several available observations to recover the main characteristics of the dynamics (its exponential ratio) and to export several dynamic and biological features. Such probabilistic-like reasoning shall be considered as complementary to formal verification techniques used for validating the qualitative properties of a system [29].

Other recent methods also use probabilistic techniques for studying gene regulatory networks [7], [9], [36]. However, their main purpose is to embed a deterministic model with probabilities. Their main analyses therefore focus on estimating impacts of variation. Probability matrices are computed to represent experiments accurately. Finally, transition probability matrices are used to compute permanent distributions. We argue that our approach is complementary since our average case analysis theory allows us to emphasize emerging properties of the system. Relations between the two scales of observations allow us to exhibit constraints between the gene regulatory network and protein observations. Eventually, this process elucidates transition probabilities that did not come to light with other available methods.

A weakness of our approach relies on the fact that the Markov Chain inference process is based on knowledge of a full qualitative gene regulatory network [4]. This shortens the range of application of our method since, nowadays, relatively few biological systems are described at this level of abstraction. However, this flaw will be moderated by the fact that the gene regulatory network is used only in order to build a global frame of the event transition Markov chain, which is much more abstracted and smaller that the gene regulatory dynamics description. It is reinforced by our main approach which is to build the Markov chain automatically from biological assumptions – either from the literature or experiments such as microarrays.

Another weakness lies in the assumption of a linear relationship between gene activity and the production of the corresponding protein (relevant for a microbial system only). To avoid such a restriction, one must build novel accumulation rules based on other biological abstractions – metabolic and environmental phenotypes are the most natural candidates here. Extending the construction of event transition Markov chain to the models containing reactions instead of qualitative regulations – for instance, signaling networks – is also under study to extend the range of application of our approach. A final range of future works relies on extracting more precise properties from the Markov chain description of a given dynamic system. Such studies shall initially focus on the interpretation of the concentration joint law, standing as a correlation coefficient between time-series observations. They will also investigate the use of these Markov chains to isolate experimental noise from the noise inherent to the chaotic properties of the system. This would provide an estimation of measurement qualities. Finally, average case analysis can be performed on a class of probabilistic models that is much larger than Markov chains. This would allow us to deal with Markov chains that may handle slight variations over the course of times, eventually studying the adaptation of the model behaviors under given environmental variations.

## Supporting Information

Text S1 Application of the method on a simple two genes example.
(PDF)

Text S2 A complete description of *Escherichia coli* model, information used during the inference task and results.
(PDF)
