# Supplementary material for: Integrating Quantitative Knowledge into a Qualitative Gene Regulatory Network
Source: PLoS Comput Biol. 2011 Sep 15;7(9):e1002157. doi: 10.1371/journal.pcbi.1002157 (PMC3174175; doi:10.1371/journal.pcbi.1002157)
Supplement: Text S1 — Application of the method on a simple two genes example. (PDF) [file pcbi.1002157.s001.pdf]

Integrating quantitative knowledge into qualitative gene regulatory network: Supplementary text S1

Application of the method on a simple two gene example

ETG modeling overview

ETG modeling approach needs two inputs: (i) a qualitative abstraction of the dynamical system, and (ii) quantitative knowledge. As results, one obtains three kinds of outputs: quantitative simulations of the dynamical system that allows (i) a validation of our results based on experimental knowledge at disposal and (ii) prediction of biological compound behaviors over times. Furthermore, sensitivity analysis emphasizes (iii) a ranking of the most important ETG transitions that must occur to satisfied the overall quantitative behavior of the system. Figure 1 pictures the modeling process overview. A MATLAB script allows to perform the ETG modeling. It can be found here[1].

Qualitative inputs of the ETG modeling

1. *Defining the ETG graph (core model):* The graph describes the qualitative behaviors of the biological regulatory network by focusing on the products of the genes. As described in the manuscript, a given gene stochastically regulated by the system either product a increase of (gene_+), or a decrease (gene_-) of its protein quantity, which characterizes what we call here two events that are related to a given gene. This graph can be manually built using biological knowledge at disposal (i.e., knowing what gene activation actives or represses what gene activations). When available, one should consider to make this graph automatically from qualitative models. For illustration, the following regulatory model of two genes formalized into a Piecewise Affine Differential Equations (PADE) system can be transformed into ETG graph in two steps:

[1] http://pogg.genouest.org

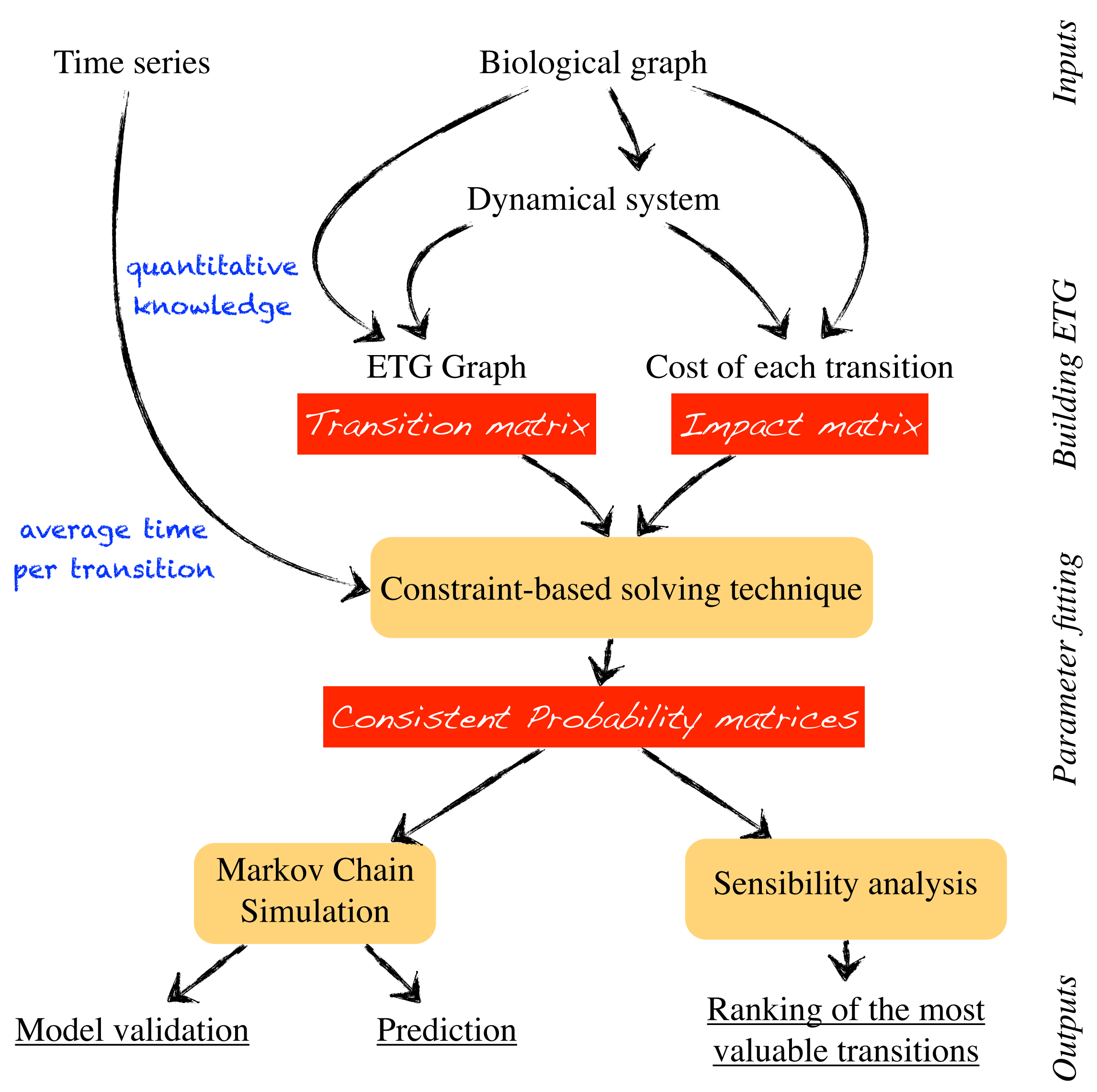

Figure 1: Flowchart of the ETG modeling process. For two given inputs, quantitative knowledge of time series and qualitative biological knowledge, the ETG modeling technique provides a simulation of the model and an automatic sensitivity analysis. Those results can be further analyzed like for validation purposes.

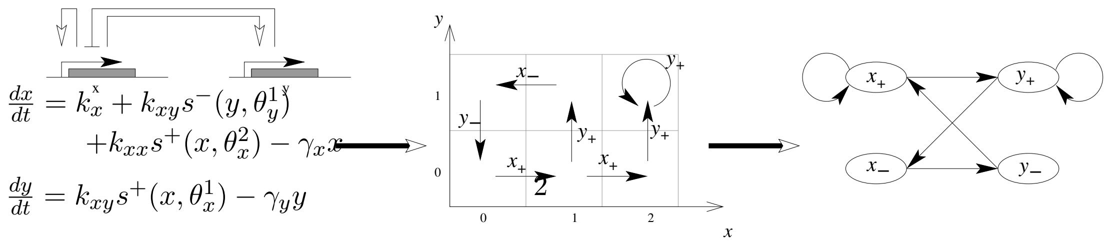

a) Two genes interaction network and its PADE definition

b) Discrete dynamics or state transition graph

c) Concentration graph

The transformation from PADE systems to ETG structures is straightforward. First, one produces a state transition graph from a PADE. It is classically obtained by fixing some inequality constraints on the parameter of the system's equations (see GNA webpage for details). Second, the ETG is made from the state transition graph by considering all possible successions of events, which is here mRNA changes. Precisely, there exists an edge from state A to state B in the ETG graph if the two successive transition paths $\stackrel{A}{\longrightarrow} \cdot \stackrel{B}{\longrightarrow}$ is present in the state transition graph.

This example is available in MATLAB. The ETG graph is described as a MATLAB matrix in `ExampleSqueleton` that defines the corresponding $\{0, 1\}$-transition matrix.

This simple example has two latent variables $v_1 = p_{x_+ \to x_+}$ and $v_2 = p_{y_+ \to y_+}$ allowing to express the unknown probability transition matrix of the model

$$\begin{pmatrix} v_1 & 1 - v_1 & 0 & 0 \\ 0 & v_2 & 1 - v_2 & 0 \\ 0 & 0 & 0 & 1 \\ 1 & 0 & 0 & 0 \end{pmatrix}$$

2. *Estimation of the impact of each transition:* we consider a cost for taking each transition of the ETG graph. The cost is a direct impact on the protein production. As a biological assumption, we assume the passive degradation rate (free parameter) to be equal to 5% for both protein X and protein Y. The value of the active production and degradation rates d_+ and d_- for protein X satisfies an equilibrium principle saying that for a uniform choice of transition probabilities, protein X expected concentration is constant. Assuming that $d_- = p$ and $d^+ = 1/p$ leads to the resolution of an equation of order 2 in p where the coefficients depend on the stationary distribution π of the transition matrix. Here, the equation is

$$1/p \; \pi_{x^+} + p \; \pi_{x^-} + 0.95 \times [1 - \pi_{x^+} - \pi_{x^-}] = 1.$$

This equation have only one solution smaller than 1, $p = 0.8818$. Thus $d_+ = 1.1341$ and $d_- = 0.8818$. The impact matrix for protein X is then

$$\begin{pmatrix} 1.1341 & 0.95 & 0 & 0 \\ 0 & 0.95 & 0.8818 & 0 \\ 0 & 0 & 0 & 0.95 \\ 1.1341 & 0 & 0 & 0 \end{pmatrix}$$

This solution is described in MATLAB format in the matrix `CostProtein_X` that describes the cost of ETG graph transitions on the protein X quantities (mainly, the transitions involved and the cost for these transitions).

Probability inference

To demonstrate the interest of our modeling approach, we consider fictive experimental data. The dataset is hence defined by the protein X concentration multiplied by 100 in 100 time unit or iterations. Assuming a multiplicative effect of the regulatory network on the protein concentrations, it corresponds to an asymptotic growth rate (observable variable of the system) of:

$$\exp(\log(100/1)/100) = 1.0471$$

One must find probabilities that allow to obtain such a growth rate. The inference of these probabilities is performed by our MATLAB script using:

```
[BS,BM]=ETG_solve(ExempleSqueleton, {CostProtein_X}, {1.0471}, confidence);
```

where `ExempleSqueleton` is the $\{0,1\}$-transition matrix and `CostProtein_X` is a structure describing the cost of protein X (mainly, the transitions involved and the cost for these transitions), `confidence` if the maximal allowed error in the numerical computations. `BM` is the matrix of probabilities that satisfy the protein growth rate, whereas `BS` is the euclidean distance between the estimated growth rate, as computed using `BM`, and the optimal growth rate as given by the experiments.

Plasticity of Event Transition Markov chain face to extreme parameters

In complementary to the results shown in the manuscript, one compares the distributions of protein Y growth rates under various conditions. All these conditions are compared to the distribution of a random case where both variables v_1 and v_2 are drawn uniformly. Then, one considers two extreme conditions for the X growth rate. For instance, this parameters may be equals to 0.97 for simulating a fast degradation of X protein, or 1.1 for simulating a major burst of protein X production. In both cases, distributions of the Y growth rate have been estimated by considering 10000 inferred probability matrices. One are then able to compute their difference of estimated Y growth rate distributions between the "random"

condition and X growth rates in both extreme conditions.

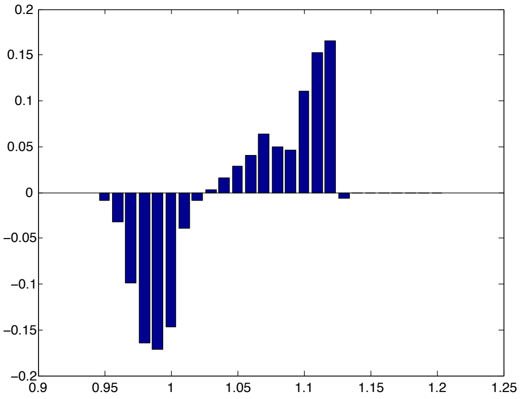

Random condition V.S. 0.97 X growth rate

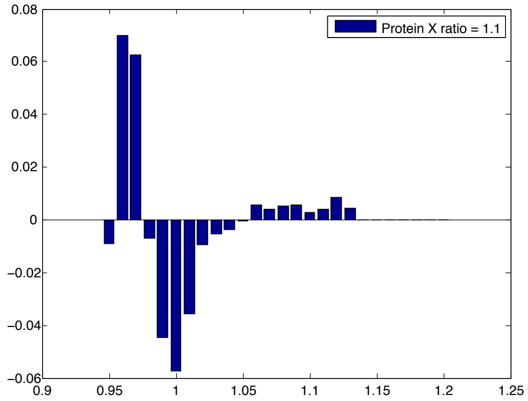

Random condition V.S. 1.1 X growth rate

The difference in the distributions highlights the inherent plasticity of the Event Transition Markov chain to estimate protein growth rates in various conditions, despite the major constraints given by the Event Transition Graph that restricts the quantitative behaviors.
