# Supplementary material for: Integrating Quantitative Knowledge into a Qualitative Gene Regulatory Network
Source: PLoS Comput Biol. 2011 Sep 15;7(9):e1002157. doi: 10.1371/journal.pcbi.1002157 (PMC3174175; doi:10.1371/journal.pcbi.1002157)
Supplement: Text S2 — A complete description of Escherichia coli model, information used during the inference task and results. (PDF) [file pcbi.1002157.s002.pdf]

Integrating quantitative knowledge into qualitative gene regulatory network: Supplementary text S2

A complete description of *Escherichia coli* model

Experimental data and model

As an application of our modeling approach, we consider the time series data extracted from:

Ball CA, Osuna R, Ferguson KC, Johnson RC (1992) Dramatic changes in Fis levels upon nutrient upshift in *Escherichia coli* *J Bacteriol* **174**: 8043–56.

and represented in Fig. 1. For sake of simplification, concentrations of both proteins are not represented by their absolute concentrations but rather converted into their respective percentages of maximal observed concentration.

times (min)	[FIS] %	[CYA] %	growth phase
0	75	75	stationary
2	10	1	stationary
30	20	1	stationary
55	50	1	stationary
70	80	1	stationary
80	100	1	exponential
100	50	100	exponential
110	30	-	exponential
130	10	130	exponential

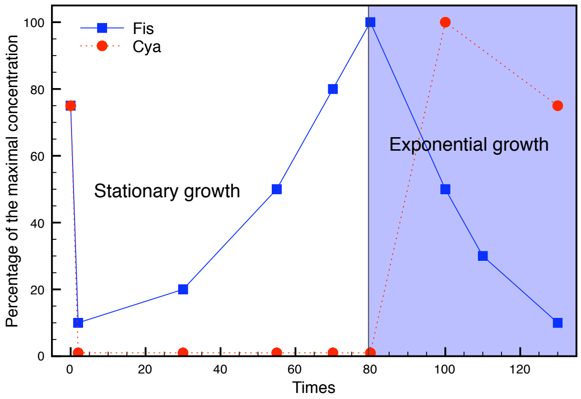

Figure 1: Experimental data at disposal

We used the biological model and corresponding biological assumptions as published in:

Ropers, D., de Jong, H. D., Page, M., Schneider, D., & Geiselmann, J. (2006). Qualitative simulation of the carbon starvation response in *Escherichia coli*. *BioSystems*, **84**(2), 124-152.

Since the model is formalized in a Piecewise Affine Differential Equation system (PADE), both its biological graph (ex: gene x activates the gene y transcription) or its formalization of the dynamical system can be used to build an ETG. As an application, we used herein the biological graph. The corresponding ETG is pictured in Figure 6 of the manuscript. Notice that the switch between the two phases impacts the event transition graph by suppressing two transitions ($fis_+ \rightarrow crp_-$ and $complex \rightarrow fis_-$ in the stationary growth phase.

Training dataset and costs

For the sake of clarity, we expose here the data used for the training the model (i.e. estimation of the probability matrices). Notice here that 3 data points are needed for finding the information.

times (min)	[FIS] %	[CYA] %	growth phase
2	10	-	stationary
80	100	-	exponential
130	10	-	exponential

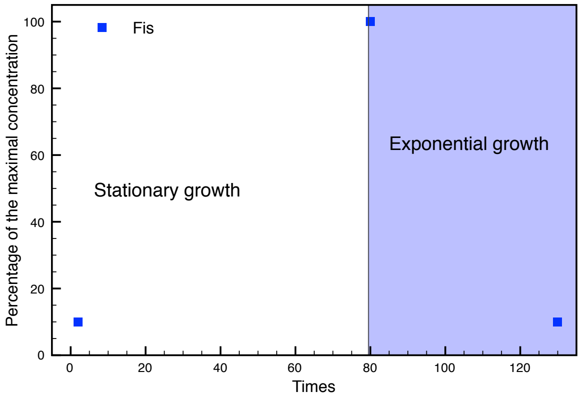

Figure 2: Experimental data used for the parameter identification

Protein costs. Following the equilibrium principle, one deduces these values for the relative protein rates.

Protein	d_+	d_-
FIS	1.4653	0.6825
CYA	2.0636	0.4846
CRP	1.4362	0.6963
TOPA	1.6181	0.6180
GYRAB	1.8662	0.5358

Datasets. The concentration evolution rates can be determined for both phases, according to Figure . For instance, the growing rate for FIS in the stationary growth phase,

computed by using is relative values at times 2 and 80 minutes, equals

$$\frac{\log(100) - \log(10)}{80 - 2} = 1.03.$$

This value says that FIS protein concentration increases by 3% each minute. In order to use it in our model, it is necessary to obtain the corresponding rate per transition of the model, and thus to know the number of iterations performed by the model in a one minute duration. We argue that FIS, CYA and other proteins are degraded as soon as a sufficient number of its amino acids are degraded. In accordance to the N-end rule [Alexander, Varshavsky (1997). "The N-end rule pathway of protein degradation". Genes to Cells 2 (1): 13-28], we take a duration of 2 minutes as the minimal half-life for these animo-acids. Thus, when taking a natural degradation rates of 5% per transition, the model runs n iterations to degrade half of the present proteins, where n satisfies $0.95^n = 0.5$. Here, $n \approx 14$ implying that 7 iterations are reached per minute. Known concentration evolution rates in both phases, expressed in a per iteration scale, are synthesized in the following table:

Protein	Stationary growth	exponential growth
FIS	1.0042[1]	0.9934[1]
CYA	0.7346[2]	1.0334[2]
CRP	?	?
TOPA	?	?
GYRAB	?	?

Mean values of the predicted concentration evolution rates in both phases (mean computed using 100 points of the solution set):

Protein	Stationary growth	exponential growth
FIS	1.0042	0.9934
CYA	0.9506	1.0745
CRP	0.9506	1.0073
TOPA	0.9819	0.9987
GYRAB	0.9700	0.9675

Figure 4 depicts an example of probabilities assignment that satisfies the expected growth ratios for protein FIS.

Model validation For the sake of validation of our modeling technique applied on E. coli, we compare the time series predicted with those observed experimentally during both

[1] used for inference
[2] used for validation

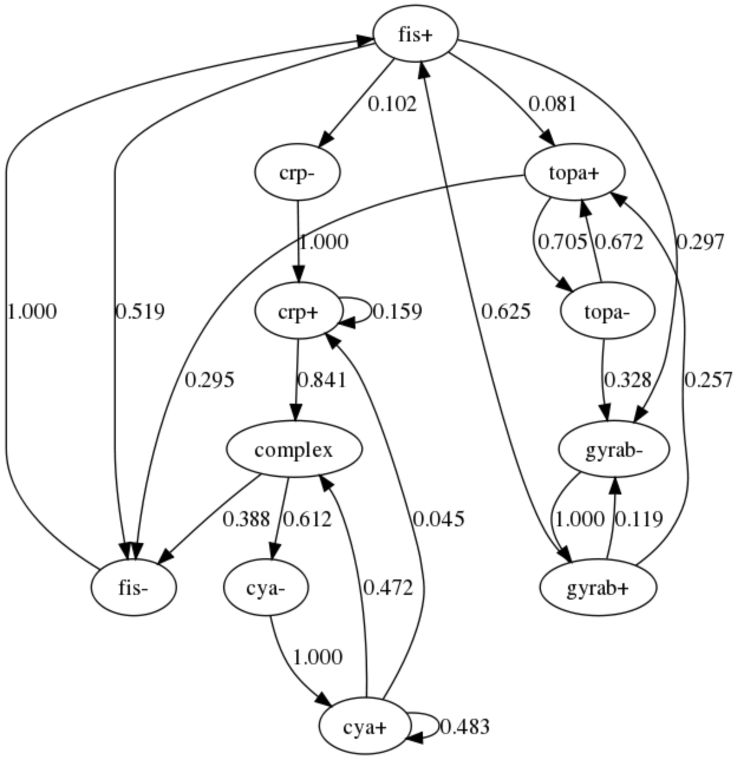

Figure 3: Event Transition graph and **an** example of corresponding probabilities after an estimation based on experimental data such as given in Figure 2. Note herein that several probabilities allow to fit the experimental knowledge.

growth phases. As previously mentioned, CYA and Fis concentration behaviors were investigated. A comparison between FIS and CYA observations and their respective predictions by the model is performed. A Pearson correlation test confirms the accuracy of the predictions. Notice that in the computed time-series, we set the value to 1 if the computed value is smaller than 1 and to 100 is the computed value is greater than 100.

Time	FIS		CYA	
	obs	pred	obs	pred
0	-	-	75	75
2	10	10	1	37
30	20	23	1	1
55	50	47	1	1
70	80	73	1	1
80	100	99	1	1
100	50	40	100	100
110	30	25	100	100
130	10	10	75	100
R^2	0.9937		0.9599	
p-value	6.5 10^{-8}		10^{-5}	

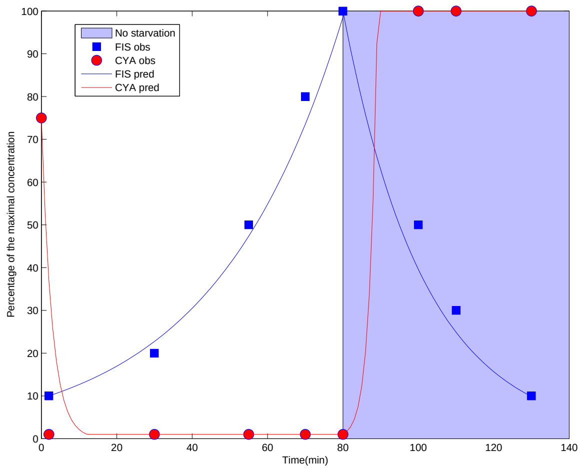

Model predictions

It is also possible to predict other concentration evolutions, assuming, for instance, an initial concentration of 50% for each unknown proteins. The resulting predictions are depicted in Figure 5.

Sensitivity of the ETG transitions

Computing the sensitivity of the model allows to rank the transitions according their partial derivative. The higher is the sensitivity of the transition, the higher it is constrained to be equal to a fixed value. It is expressed in percentage having the following meaning: if the given probability is changed by 1%, then the euclidean distance between the expected growth ratio and their predictions is modified by $X\%$ (the given sensitivity). Each returned sensitivity is computed as the mean over 100 transition matrices satisfying FIS observed protein evolution. Such an information is useful to classify the transitions according to their importance on the system. The sensitivities are depicted in Figure 6.

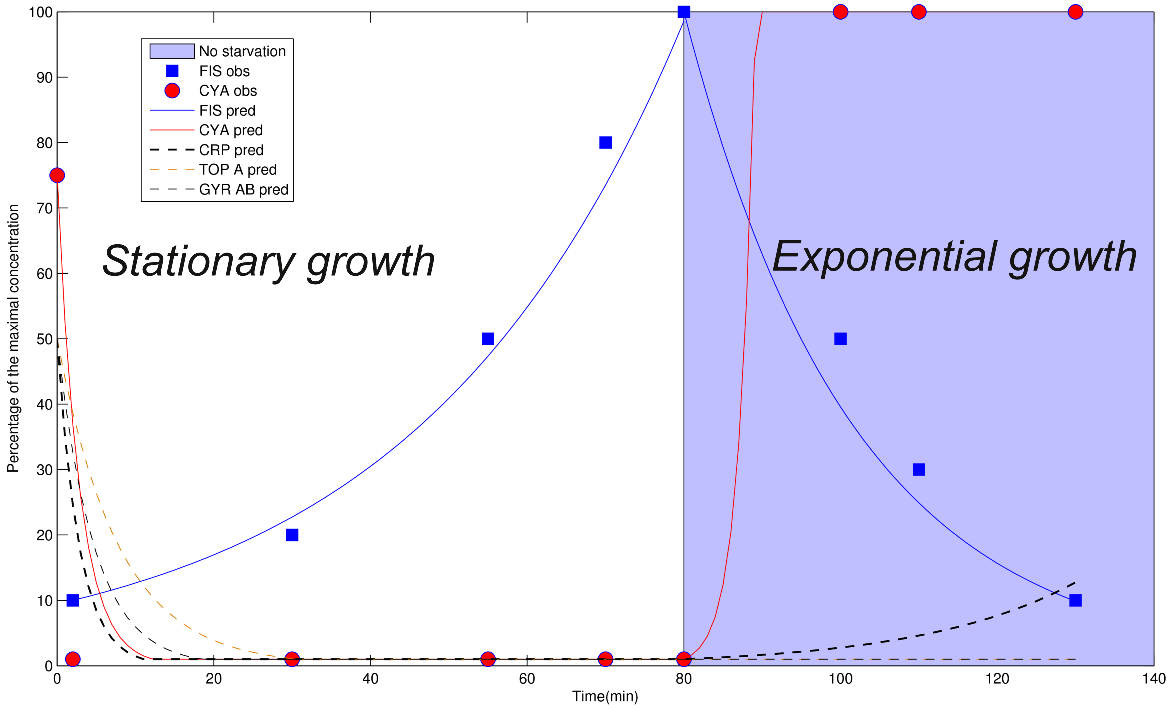

Figure 4: Predictions of diverse protein concentrations related to the studied system.

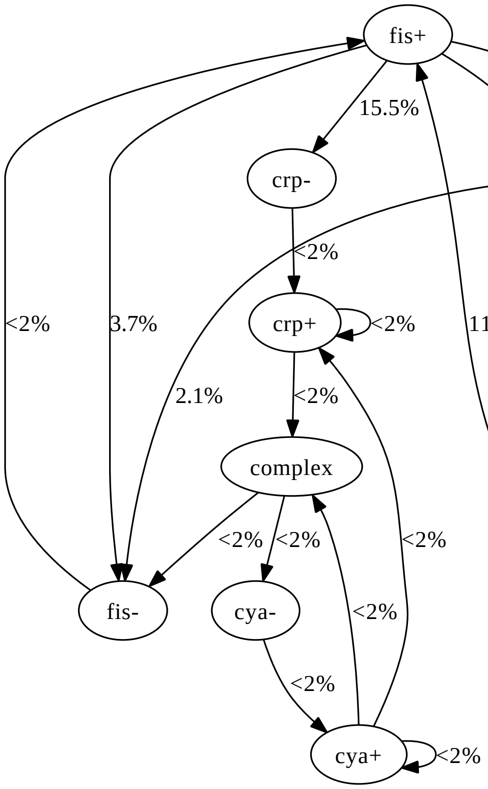

Figure 5: Event Transition graph and corresponding sensitivities after an estimation based on experimental data such as given in Figure 2
